# Recent Emergence and Spread of an Arctic-Related Phylogenetic Lineage of Rabies Virus in Nepal

**DOI:** 10.1371/journal.pntd.0002560

**Published:** 2013-11-21

**Authors:** Ganesh R. Pant, Rachel Lavenir, Frank Y. K. Wong, Andrea Certoma, Florence Larrous, Dwij R. Bhatta, Hervé Bourhy, Vittoria Stevens, Laurent Dacheux

**Affiliations:** 1 Rabies Vaccine Production Laboratory, Tripureshwor, Kathmandu, Nepal; 2 Institut Pasteur, Unit Lyssavirus Dynamics and Host Adaptation, National Reference Centre for Rabies, WHO Collaborating Centre for Reference and Research on Rabies, Paris, France; 3 Australian Animal Health Laboratory, CSIRO Animal Food and Health Sciences, Geelong, Victoria, Australia; 4 Central Department of Microbiology, Tribhuvan University, Kirtipur, Kathmandu, Nepal; University of Minnesota, United States of America

## Abstract

Rabies is a zoonotic disease that is endemic in many parts of the developing world, especially in Africa and Asia. However its epidemiology remains largely unappreciated in much of these regions, such as in Nepal, where limited information is available about the spatiotemporal dynamics of the main etiological agent, the rabies virus (RABV). In this study, we describe for the first time the phylogenetic diversity and evolution of RABV circulating in Nepal, as well as their geographical relationships within the broader region. A total of 24 new isolates obtained from Nepal and collected from 2003 to 2011 were full-length sequenced for both the nucleoprotein and the glycoprotein genes, and analysed using neighbour-joining and maximum-likelihood phylogenetic methods with representative viruses from all over the world, including new related RABV strains from neighbouring or more distant countries (Afghanistan, Greenland, Iran, Russia and USA). Despite Nepal's limited land surface and its particular geographical position within the Indian subcontinent, our study revealed the presence of a surprising wide genetic diversity of RABV, with the co-existence of three different phylogenetic groups: an Indian subcontinent clade and two different Arctic-like sub-clades within the Arctic-related clade. This observation suggests at least two independent episodes of rabies introduction from neighbouring countries. In addition, specific phylogenetic and temporal evolution analysis of viruses within the Arctic-related clade has identified a new recently emerged RABV lineage we named as the Arctic-like 3 (AL-3) sub-clade that is already widely spread in Nepal.

## Author Summary

Rabies is endemic in most Asian countries and represents a serious public health issue, with an estimated 31,000 people dying each year of this disease. The majority of human cases are transmitted by domestic dogs, which act as the principal reservoir host and vector. However, molecular epidemiology and evolutionary dynamics of the main etiological agent, the rabies virus (RABV), remains largely unappreciated in some regions such as in Nepal. Based on a subset of 24 new Nepalese isolates collected from 2003 to 2011 and representative RABV strains at a global scale, phylogenetic analysis based on the complete nucleoprotein and glycoprotein genes sequences revealed the presence of a surprising wide genetic diversity of RABV circulating in this country. The presence of three different co-existing phylogenetic groups was identified: an Indian subcontinent clade and two different Arctic-like sub-clades within the Arctic-related clade, namely Arctic-like (AL)-1, lineage a (AL-1a), and AL-3. Among these clusters, the AL-3 sub-clade appears as the major Nepalese phylogroup which emerged relatively recently in this country, within the last 30 years. These data has raised some concerns about the exchange of RABV between different countries, and provided key elements for implementation of effective control measures of rabies in Nepal.

## Introduction

*Rabies virus* (RABV) is the prototype species of the genus *Lyssavirus*, in the family *Rhabdoviridae* [1]. Members of this viral genus encompass single-stranded, negative sense viruses with a genome size of nearly 12 kb. Among the 12 species of lyssaviruses identified to date, RABV has the broadest geographic distribution and the widest spectrum of vectors or reservoir hosts within the orders Carnivora and Chiroptera [2], [3]. This zoonotic virus remains the main etiological agent of rabies, an acute and almost invariably fatal form of encephalomyelitis, which can affect almost all terrestrial mammals, including humans. Infection occurs after contamination with infected saliva by bites, scratches and mucous membrane exposure. Despite the availability of an effective post-exposure prophylaxis, it is estimated that approximately 55,000 people die every year due to rabies, with more than 95% of human deaths occurring in Asia and Africa [4]. To date, no effective treatment exists when clinical disease is declared [3].

The domestic dog remains the main reservoir and vector of rabies in developing countries, and is responsible for almost all human deaths. Various wild carnivores are also involved in the maintenance of RABV and transmission of sylvatic rabies in limited geographic regions, with a small contribution in the burden of human rabies. Other terrestrial mammal species including livestock species are susceptible to rabies but do not transmit the disease further, acting as epidemiological dead-end hosts [2].

The dog has also been identified as the probable main vector involved in interspecies RABV transmission. Indeed, phylogeographic analyses indicated that current RABV from non-flying mammals cluster into six major geographically distinct clades with a strong indication that these clades share a common ancestor originating from domestic dogs in the southern parts of the Indian subcontinent, and that the subsequent evolutionary diversification probably occurred within the last 1500 years [5]. This study and others highlight the important role that improved knowledge of the biodiversity of the disease and especially potential routes of spread play in helping to design control and prevention strategies [5]–[7].

However, the global burden of rabies remains largely underestimated in most of enzootic areas, particularly in Africa and Asia [4], [8], [9]. The real incidence of human rabies is poorly documented, and data related to the epidemiological cycle of rabies in domestic and wildlife animal populations in some Asian countries are spare. This is particularly true for Nepal, where rabies is endemic. Incidence of human rabies is estimated to 100–200 documented deaths every year, but most of the cases are diagnosed on clinical signs and history of dog bite, and rarely confirmed by laboratory tests. In 2010, a total of 41,200 people received free post-exposure prophylaxis, a tissue culture inactivated rabies vaccine [10], [11]. Hyperimmune serum (horse and human) against rabies is only available in Kathmandu city. Surveillance and prevention of animal rabies remain limited; in 2010, administration of 12,426 vaccinations to animals was conducted [12]. Meanwhile the Veterinary Epidemiology Centre in Kathmandu recorded 143 animal deaths for the entire country during the same period, but laboratory diagnosis of rabies was only made on a limited portion of these at the Central Veterinary Laboratory (CVL) in Kathmandu due to difficulties in transportation of clinical samples from the countryside. In the absence of an active national surveillance plan and difficulties in sample transport, passive rabies surveillance often occurs only when a dead and/or infected animal is brought to the CVL. Therefore information and characterization of representative viruses from Nepal remain limited. A better understanding of the dynamics of RABV circulation and potential exchanges with neighboring countries is necessary and valuable for future control plans and prevention strategies. In this study, we performed the molecular characterisation of RABV isolates circulating in Nepal, based on the complete nucleoprotein (N) and glycoprotein (G) genes sequences of new isolates. We also compared the study isolates to representative viruses from other regions to place them in an international context. The combined database of rabies virus sequences has yielded insights into their phylogeography and enabled a specific examination of the timeframe of emergence of the Arctic-related clade, allowing the identification of a new and emerging Nepalese phylogenetic group of RABV.

## Methods

### Ethics statement

The human clinical sample described in this paper was collected in 2003 and following informed oral consent from the child's parents, was sent to the National Reference Centre for Rabies housed in the Lyssavirus Dynamics and Host Adaptation (LDHA) Unit, Institute Pasteur in 2009. This sample had been registered for research purpose in the LDHA Unit biobank and declared according to the French regulations (article L.1243-3 in relation to the French Public Health Code) to both the French Ministry for Research and a French Ethics Committee which both approved and registered the biobank (declaration number DC-2009-1067; collection N°12).

### Description of RABV isolates

Twenty-four clinical rabies samples were collected from seven districts in Nepal between 2003 and 2011 and included domestic dogs (n = 14), livestock with goat (n = 4), cattle (n = 3) and buffalo (n = 1), and 1 each of human and mongoose (Table 1 and Table S1). These samples were collected either at the Regional Veterinary Laboratory or CVL from dead animals that had shown clinical signs prior to death. These samples were tested at the CVL for rabies confirmation by a rapid antigen detection and fluorescent antibody test (FAT) [13]. As part of this study, all confirmed rabies positive samples were shipped as either fresh frozen tissue or dried tissue smears on FTA Cards (Whatman, USA) to the Australian Animal Health Laboratory or Institut Pasteur, Paris (IPP) for further phylogenetic analyses (Table 1). One human sample originated from a 12 year-old girl who had passed away after being bitten by a dog in 2003 [14]; this sample was analysed at IPP. All samples were subjected to molecular characterization, and a viable subset was also used for virus isolation.

**Table 1:** Description of RABV samples sequenced in this study.

Isolate	Type of sample	Host species	Origin (region or city)	Date of collection	GenBank accession no.	
					N gene	G gene
4403-12	Vaccine	Vero cells	-	-	JX944594	JX944575
4403-13	Brain	Human	Nepal (Lalitpur region)	30/07/03	JX944565	JX944584
4403-14	Brain	Dog	Nepal (Kathmandu region)	02/11/08	JX944595	JX944576
4403-16	Brain	Dog	Nepal (Dhading region)	20/02/09	JX944596	JX944577
4403-17	Brain	Dog	Nepal (Kaski region)	09/11/09	JX944597	JX944578
4403-19	Brain	Dog	Nepal (Lalitpur region)	13/11/09	JX944598	JX944579
3878-02	Brain	Dog	Nepal (Kathmandu region)	11/04/10	JX944566	JX944585
3878-03	Brain	Dog	Nepal (Kathmandu region)	21/04/10	JX944599	JX944580
3878-04	Brain	Dog	Nepal (Bhaktapur region)	04/02/10	JX944600	JX944581
3878-05	Brain	Mongoose	Nepal (Kaski region)	20/05/10	JX944601	JX944582
3878-08	Brain	Dog	Nepal (Kathmandu region)	03/09/10	JX944602	JX944583
3878-09	Brain	Dog	Nepal (Kathmandu region)	23/08/10	JX944567	JX944586
3878-10	Brain	Dog	Nepal (Kathmandu region)	30/09/10	JX944568	JX944587
3878-73	Brain (FTA card)	Cattle	Nepal (Kaski region)	28/05/09	JX944569	JX944588
3878-74	Brain (FTA card)	Goat	Nepal (Tanahu region)	03/06/10	JX944570	JX944589
3878-75	Brain (FTA card)	Goat	Nepal (Kaski region)	16/06/10	JX944571	JX944590
3878-76	Brain (FTA card)	Cattle	Nepal (Kaski region)	25/02/10	JX944572	JX944591
3878-77	Brain (FTA card)	Goat	Nepal (Bhaktapur region)	27/06/10	JX944573	JX944592
3878-78	Brain (FTA card)	Goat	Nepal (Makwanpur region)	28/08/10	JX944574	JX944593
09029NEP	Brain	Buffalo	Nepal (Kaski region)	11/07/03	JX987737	JX987721
09031NEP	Brain	Dog	Nepal (Kathmandu region)	08/09/08	JX987738	JX987722
11001NEP	Brain (FTA card)	Cattle	Nepal (Lalitpur region)	12/11/09	JX987740	JX987724
11015NEP	Brain (FTA card)	Dog	Nepal (Kathmandu region)	02/01/11	JX987741	JX987725
11016NEP	Brain (FTA card)	Dog	Nepal (Kathmandu region)	24/01/11	JX987742	JX987726
11017NEP	Brain (FTA card)	Dog	Nepal (Kathmandu region)	05/02/11	JX987743	JX987727
04035AFG	Brain	Dog	Afghanistan (Kabul city)	2004	GU992304	JX987720
02052AFG	Brain	Dog	Afghanistan (Kabul city)	2002	JX987735	JX987718
04027AFG	Brain	Dog	Afghanistan (Kabul city)	1996	EU086162	JX987719
04029AFG	Brain	Dog	Afghanistan (Kabul city)	2004	JX987736	EU086128
09032AFG	Brain	Dog	Afghanistan (Kabul city)	2009	JX987739	JX987723
9319IRA	Brain	Jackal	Iran (Kashmar city)	-	JX987746	JX987732
96321IRA	Brain	Jackal	Iran (Shahrud city)	1996	JX987748	JX987734
8681IRA	Brain	Dog	Iran (Tehran city)	1985	U22482	JX987728
8682IRA	Brain	Sheep	Iran (Shiraz city)	1974	JX987744	JX987729
9141RUS	Brain	Arctic fox	Russia (Yakutia region)	1988	U22656	-
9143RUS	Brain	Arctic fox	Russia (Yakutia region)	1988–90	JX987747	JX987733
8683GRO	Brain	Dog	Greenland	1980	JX987745	JX987730
8684GRO	Brain	Arctic fox	Greenland	1981	U22654	JX987731

Additionally, a number of new or partially characterized isolates from other countries were also included in this study, originating from Afghanistan (n = 5), Greenland (n = 2), Iran (n = 4), Russia (n = 2) and USA (n = 1). Host animal origins and dates of collection are indicated in Table 1 and Table S1, when available. In addition, a vaccine strain used in Nepal and historically supplied by IPP was also analysed in this study.

### Virus isolation

Brain samples received by the Australian Animal Health Laboratory were processed for virus isolation. Briefly, a 10% homogenate of each brain was prepared in PBSA pH 7.4, using a blunt end 18 g needle and syringe in a class II cabinet. Cellular debris was removed by centrifugation at 1000 g for 3 min at room temperature. The brain homogenate (200 µl) was added to Neuro-2a cells in HMEM medium with 10% fetal calf serum in a 25 cm^2^ flask. The cell culture was incubated for 4–7 days at 37°C in a CO_2_ incubator and regularly inspected for evidence of cytopathic effect. Cells with evidence of cytopathic effect were removed from the flask and harvested by centrifugation.

### Extraction of RNA

Total RNA was extracted using either Magmax Viral Isolation Kit (Applied Biosystems, Foster City, USA) or Tri-Reagent (Molecular Research Center, Inc., Cincinnati, USA), both according to the manufacturer's instructions. Extraction of RNA was performed from the original brain samples, from cell suspension after virus isolation or from FTA cards. For the latter, RNA recovery was achieved from hole-punched card material according to manufacturer's instructions or after incubation for 30 min in 100 µl Ambion RNA Rapid Extraction Solution (Applied Biosystems, Foster City, USA).

### RT-PCR and sequencing

RT-PCR amplification and DNA sequencing of the N and G genes were performed as described previously [5], [15]–[19]. Analysis of sequence data and contig assembly were performed using either the SeqMan module of the Lasergene v8.0 software suite (DNASTAR) or Sequencher 5.0 (Gene Codes Corporation) software. GenBank accession numbers for the complete N and G gene sequences newly acquired in this study are designated JX944565-JX944602 and JX987718-JX987749.

### Phylogenetic analysis

Complete N and G gene coding sequences determined in this study were analysed with respective full-length sequence datasets comprising rabies N gene sequences (n = 173, 1350 nt) and G gene sequences (n = 92, 1575 nt) available from GenBank (Table S1). Multiple sequence alignments and amino acid residue analysis were performed using Clone Manager v.9 (Science & Educational software) or ClustalX 2.1 [20]. Maximum-likelihood (ML) phylogenies were inferred for each dataset using the GARLI v2.0 [21] and PAUP* v4.0 programs [22]. The general time reversible model with proportion of invariable sites plus gamma distributed rate heterogeneity (GTR+I+ Γ4) as generated by the Akaike information criterion using the program MODELTEST v3.7 [23], was used as the best-fit nucleotide substitution model in all cases. Reliability of the ML tree topologies was tested using both neighbour-joining (NJ) and ML methods with 1,000 and 100 bootstrap replicates respectively.

### Evolutionary analysis

Using a select RABV Arctic-related clade dataset of 67 N gene sequences, we inferred a maximum clade credibility (MCC) tree by using the Bayesian Markov chain Monte Carlo (MCMC) method available in the BEAST package [24]. Sequences were dated with the year of isolation, and identical sequences with the same year were excluded. The alignment used is available from the authors on request. Posterior probability values provide an assessment of the degree of support for each node on the tree. This analysis utilized the GTR+I+Γ4 model of nucleotide substitution. All chains were run for a sufficient length to ensure convergence, with 10% removed as burn-in. After comparison of the log likelihood values calculated for several runs through the TRACER program (http://tree.bio.ed.ac.uk/software/tracer/), a relaxed (uncorrelated exponential) molecular clock was the best supported under Bayes Factors. As well as generating the MCMC tree, this analysis also allowed us to estimate both the rate of nucleotide substitution per site (substitutions per site per year) and the time to the most recent common ancestors (TMRCA) in years. The degree of uncertainty in each parameter estimate was provided by 95% highest posterior density (HPD) values. Assignment of lineages used for the description of the different phylogroups described in this study has been previously defined at the clade level [5] and within the Arctic-related sub-clades [25].

## Results

### Phylogeographical diversity of RABV in Nepal

**Figure 1:**
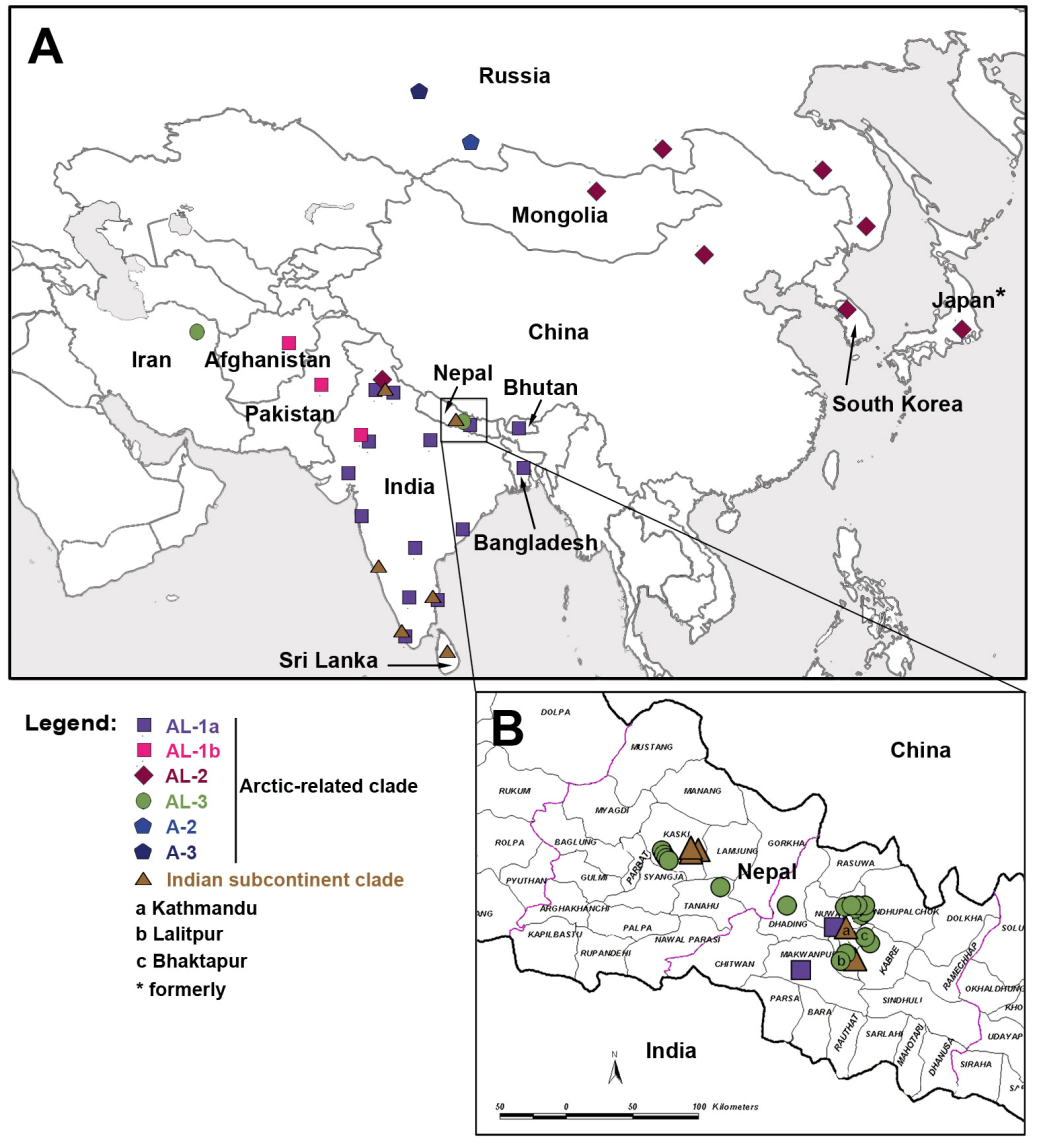
A: Distribution of the Arctic-related and Indian subcontinent phylogenetic clades of rabies virus in Asia. This map was achieved after compilation of data generated in this study and from previous reports [25], [26], [28]–[30], [35], [38], [53]–[57]. B: Detailed map of a selected region in Nepal showing the distribution of Nepalese isolates collected in this study.

The 24 Nepalese RABV specimens received for this study were collected during the period 2003–2011 from seven districts in Nepal, all classified as hill terrains and localized in the Central and Western regions of the country (Table 1, Table S1 and Figure 1). All except two of the samples from Nepal have been collected from dogs or livestock.

**Figure 2:**
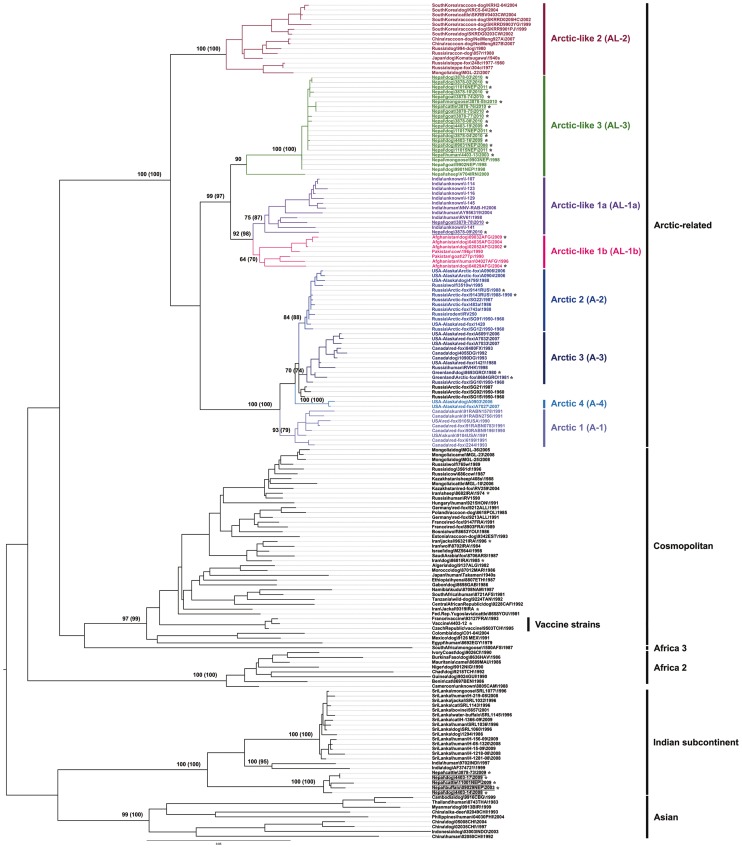
Maximum likelihood phylogenetic tree based on 173 complete RABV N gene nucleotide sequences. Branches are labeled with bootstrap values generated with both the neighbor-joining (1,000 bootstrap replications) and maximum likelihood (100 bootstrap replications, number in brackets) algorithms. Bootstrap values are given for selected relevant nodes only. A scale indicating genetic distance is presented by the horizontal bar. Designations of the different RABV phylogroups (clade, sub-clade and lineage) are as indicated, based on previously defined assignments [5], [25]. Isolates for which the complete N gene sequence was obtained in this study are indicated by an asterisk with the viruses from Nepal underscored.

**Figure 3:**
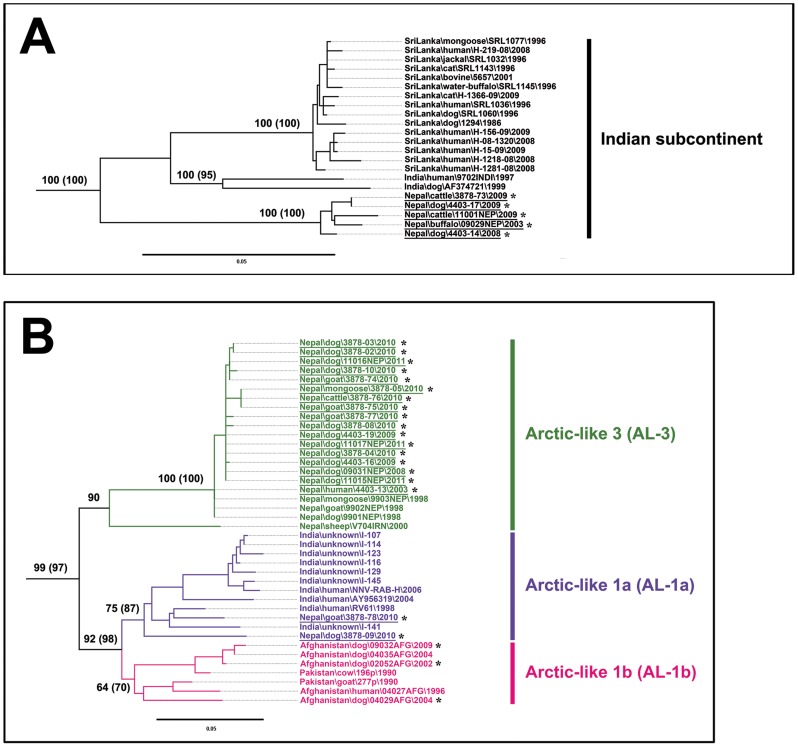
A: Magnified phylogeny of the Indian subcontinent clade. B: Magnified phylogeny of the Arctic-like 1 and 3 sub-clades. Branches are labeled with bootstrap values generated with both the neighbor-joining (1,000 bootstrap replications) and maximum likelihood (100 bootstrap replications, number in brackets) algorithms. Bootstrap values are given for selected relevant nodes only. A scale indicating genetic distance is presented by the horizontal bar. Designations of the different RABV phylogroups (clade, sub-clade and lineage) are as indicated, based on previously defined assignments [5], [25]. Isolates for which the complete N gene sequence was obtained in this study are indicated by an asterisk with the viruses from Nepal underscored.

Both NJ (data not shown) and ML (Figure 2) trees of N gene sequences exhibited similar clustering patterns, each with strong bootstrap support. In particular, all these newly sequenced specimens from Nepal were grouped into two of the six main phyloclades of canine rabies previously defined (i.e. the Indian subcontinent, Asian, Africa 2, Africa 3, Cosmopolitan and Arctic-related clades) [5]. No correlation between the geographical district source and host of the isolates to clade designation was apparent. Five isolates (4403-14, 4403-17, 3878-73, 09029NEP and 11001NEP) clustered into the Indian subcontinent clade, previously only occupied by viruses from Sri-Lanka and India (mainly from southern regions for the latter) (Figures 2 and 3A). Within this clade, viruses grouped with a clear separated spatial structure according to their geographical origin. The other 19 specimens clustered within the Arctic-related clade, which exhibited three distinct sub-clades as previously defined, the first being the Arctic sub-clade subdivided into four lineages A-1, A-2, A-3, and A-4 [25]–[27]. The two other sub-clades are represented by the Arctic-like 2 sub-clade (AL-2), and the Arctic-like 1 (AL-1) sub-clade, split into the AL-1a and AL-1b lineages, and [25]–[27]. Two of the Nepalese viruses (3878-09 and 3878-78 collected in 2010) were clustered into the AL-1a lineage, with viruses from India (Figures 2 and 3B). The remaining isolates (n = 17) formed a new and strongly supported monophyletic group, which was designated as the Arctic-like 3 (AL-3) sub-clade (Figures 2 and 3B). These isolates had 99–100% pairwise nucleotide sequence similarity to each other and to isolate 9901NEP, a virus that was isolated in Nepal from a dog in 1998 [5]. Isolates in the AL-3 sub-clade displayed 94–95% and only 86–87% pairwise nucleotide sequence similarities compared with Nepalese viruses in the AL-1a sub-clade and the Indian subcontinent clade, respectively. A single RABV isolate from Iran (V704IRN) [28] was found to be the only previously sequenced non-Nepalese representative of AL-3. This virus had 95% pairwise nucleotide sequence similarity to the viruses from Nepal, suggesting a geographical clustering.

The translated nucleoprotein sequence revealed 99–100% sequence similarity for all Arctic-like sub-clade viruses, regardless of country of origin, whilst the Indian subcontinent clade viruses displayed at least 2–4% protein sequence dissimilarity to the Arctic-like viruses. Several positions with distinctive amino acid differences were observed for the Nepalese viruses between these two major N gene phylogroups (Indian subcontinent and Arctic-related clades) which could potentially be useful as clade or sub-clade markers (Table 2).

**Table 2:** Clade specific amino acid differences in the nucleoprotein of Nepalese RABV.

Position	Type of amino acid	
	Indian subcontinent clade	Arctic-related clade
60	Met	Leu
84	Ser	Thr
110	Asp	Glu
135	Ser	Pro
179	Val	Ile^a^
332	Ala	Gly
371	Ala	Thr
379	Ile	Val
407	Thr	Ala

a Excludes virus sample 3878-10 which adopts Val at position 179.

Based on complete N sequences analysis of all available RABV viruses from Nepal, all were phylogenetically relatable to canine rabies regardless of lineage. In particular, virus of two samples collected from mongoose, 3878-05 and 9903NEP were indistinguishable from viruses originating from dogs or livestock, and could probably be from spill-over transmissions of canine rabies.

In addition to the Nepalese isolates, phylogenetic analysis of the N gene sequences in this study showed that viruses from Afghanistan (n = 5) clustered with RABV isolates from Pakistan into the AL-1b lineage (Figures 2 and 3B). Isolates from Iran (n = 4) were found to be grouped in the Cosmopolitan clade (Figure 2). The other new isolates belonged to the Arctic sub-clade, grouped in the lineage A-1 for isolates 9104USA and 9105USA, the lineage A-2 for 9141RUS and 9143RUS, and the lineage A-3 for 8683GRO and 8684GRO. The vaccine reference strain (4403-12) used in Nepal and historically originated from IPP grouped with other vaccine viruses within the Cosmopolitan clade (Figure 2).

Phylogenetic NJ (data not shown) and ML (Figure S1) trees of complete G gene sequences confirmed the results obtained with N gene. Despite the smaller G gene sample size, similar topologies were found, indicating that the distribution of Nepalese RABV within both Indian subcontinent (n = 5) and Arctic-related (n = 19) clades are defined equivalently with strong bootstrap support by both N and G gene phylogenies. Clustering patterns of the non-Nepalese RABV were also similar between N and G gene trees. The translated glycoprotein sequences showed that the Nepalese AL-3 viruses had 99–100% amino acid sequence similarity with each other, 97–98% sequence similarity to the Nepalese viruses from AL-1a, and 94% sequence similarity to the Nepalese viruses within the Indian subcontinent clade. As for N gene, positions of distinctive amino acid differences were identified between the Indian subcontinent and Arctic-related G gene phylogroups (Table 3).

**Table 3:** Clade specific amino acid differences in the glycoprotein of Nepalese RABV.

Position^a^	Type of amino acid	
	Indian subcontinent clade	Arctic-related clade
83	Arg	Lys
102	Leu	Met
156	Gly	Ser
193	Val	Thr
204	Gly	Ser^b^
231	Pro	Leu
241	Ala	Val
243	Ile	Met
426	Leu	Gln
436	Ser	Asn^b^
443	Met	Val
449	Thr	Val
450	Ala	Val
454	Met	Ala^c^
457	Leu	Ile
462	Arg	Gly
464	Gly	Val
466–468	Gln-Ala-Gly	Arg-Pro-Lys
470	Lys	Thr
473	Gly	Ser^d^
487	Asn	Ser

a Amino acid positions exclude the 19aa signal peptide sequence.

b Virus samples 3878-09 and 3878-78 have the Indian subcontinent residue.

c Virus samples 3878-09 and 3878-78 have Thr substitution.

d Virus sample 3878-78 has the Indian subcontinent residue.

### Regional evolution of RABV Arctic-related clade

**Figure 4:**
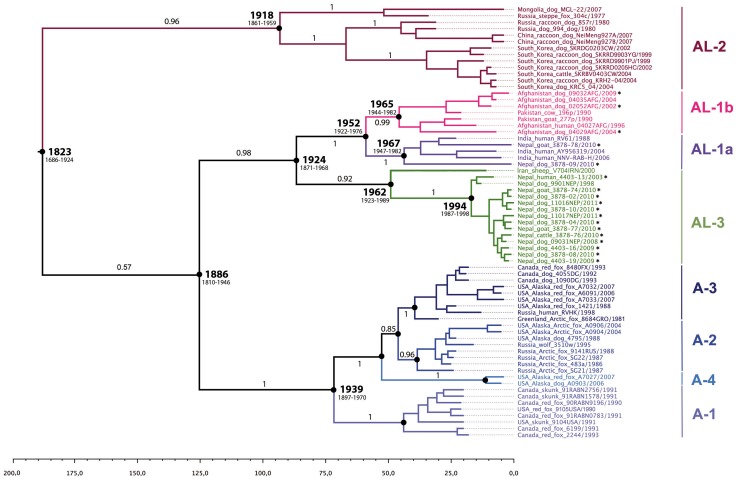
Maximum clade credibility phylogenetic tree based on 67 Arctic-related RABV N gene sequences. The horizontal branches are drawn to a scale of estimated year of divergence. Upper and lower limits of the 95% highest posterior density (HPD) estimates, and the corresponding divergence dates for the selected nodes are shown. Posterior probability values are shown above the branch for relevant key nodes only. Isolates for which the complete N gene sequence was obtained in this study are indicated by an asterisk.

Using Bayesian approaches, MCC trees were produced based on data subsets comprising 67 complete N gene sequences (Figure 4) to further clarify the relationships among the Arctic-related clade. Four major clusters were identified with good Bayesian posterior probability support, the Arctic and the Arctic-like sub-clades AL-1, AL-2 and AL-3. The time of most recent common ancestor (TMRCA) for the Arctic-related clade was estimated around 1823 (95% HPD 1686–1924). The AL-2 sub-clade, regrouping isolates from China (Inner Mongolia), Mongolia, Russia (Siberia), and South Korea appeared to be first evident around the year 1918 (95% HPD 1861–1959) (Figure 4). The subdivision between AL-1 and AL-3 sub-clades appeared around the year 1924 (95% HPD 1871–1968), leading to their emergence in 1952 (95% HPD 1922–1976) and 1962 (95% HPD 1923–1989), respectively. The AL-1 sub-clade later diverged into two lineages, AL-1a (TMRCA 1967, 95% HPD 1947–1982) that encompassed isolates from India and Nepal, and AL-1b (TMRCA 1965, 95% HPD 1944–1982) that included viruses from Afghanistan and Pakistan. Subdivision of the AL-3 sub-clade into two branches was also clearly evident, one represented only by a single Iranian isolate collected in the far east of the country, and a second distinct branch encompassing only RABV isolates from Nepal with TMRCA estimated at 1994 (95% HPD 1987–1998). The four Arctic lineages (A-1, A-2, A-3, and A-4) evolved from a common progenitor that dated back to around 1939 (95% HPD 1897–1970) (Figure 4).

The mean rate of nucleotide substitution estimated among the Arctic-related clade was 3.8×10^−4^ per site per year (95% HPD, 2.3–5.4×10^−4^ per site per year), which is in agreement with previously determined rate for the overall RABV species [5].

## Discussion

This study provides for the first time a comprehensive analysis of the genetic diversity of RABV circulating in Nepal. Despite Nepal possessing a relatively small land area (800×200 km, 147,181 km^2^) represented by a huge variety of landscapes ranging from tropical humid regions in the south to the world's highest mountains to the north (with the presence of eight mountain summits among the ten highest in the world), a high diversity of RABV was found and a rapid spread of new variants were identified in this country. Indeed, two RABV phylogenetic clades have been identified in Nepal, the Indian subcontinent and the Arctic-related clades as well as the presence of two Arctic-related sub-clades AL-1 and AL-3.

The Indian subcontinent RABV clade contains phylogenetically related viruses from Sri Lanka and India, in addition to the Nepalese isolates described in our study [29]–[33]. Due to the limited number of representative sequences, the relevant time-frames of emergence of the Indian subcontinent clade and its subsequent geographical diversification were not further determined in our study. A previous study based on the complete N gene sequences of several Sri Lankan viruses and only one Indian isolate, suggested that both diverged from their common ancestor around the year 1854 (95% HPD 1760–1918) [31]. However, the large time range associated with this estimation indicates that this result clearly needs to be confirmed on a larger dataset of sequences, which should include Nepalese viruses. Previous description of a single Nepalese RABV isolate V120 suggested that this lineage was already present in Nepal in 1989 [34]. Our characterization of a further five viruses from Nepal isolated between 2003 and 2009 has confirmed that this distinct regional sub-group of Indian subcontinent clade viruses have circulated in Nepal for at least the last 24 years.

The RABV Arctic-related clade has been relatively well described with characterization of RABV from Russia, Alaska and India in recent studies [25], [26], [35]. This clade emerged and spread relatively recently, with the most recent common ancestor dated back approximately 200 years ago [26]. This Arctic-related clade includes the so-called Arctic and the Arctic-like sub-clades.

Although RABV belonging to the Arctic-like sub-clade was already known to circulate in Nepal [5], [35], this study has identified for the first time the presence of two Arctic-like sub-clades of RABV (AL-1 and AL-3) in this country. Within the AL-1 sub-clade, we identified two Nepalese isolates belonging to the AL-1a lineage, which encompassed virus strains from India but also probably from Bangladesh and Bhutan, although for the latter phylogenetic information contained in only partial N gene sequences available in public databases was not informative enough to classify strains into either AL-1a or AL-1b lineages (data not shown) [36], [37]. AL-1a viruses have been present in India, which forms the largest immediate border with Nepal, since at least 1988 and represent currently the main phylogroup of RABV circulating in the country [26], [38] (Figures 1, 2 and S1). However, without further representation, it is unknown whether AL-1a is also an established and stable lineage in Nepal. Within this cluster, viruses originating from Nepal and India were interspersed, suggestive of the existence of movement of RABV infected domestic animals between these countries. This adds to evidence suggesting the existence of a degree of long-distance, human mediated transborder dissemination of RABV as previously also reported in Africa and hypothesized in some South East Asian countries [7], [16], [37], [39], [40]. The high prevalence of rabies in India has been suggested to be an important source of rabies outbreaks into neighboring regions [26]. Indeed, trade exchanges take place between Nepal and India, in addition to movement of people for tourism or religious purposes. The second lineage AL-1b included interspersed isolates originated from Afghanistan and Pakistan. A single Indian isolate collected from Jodhpur had also been associated to this cluster, reflecting a probable translocation of this virus from the Pakistani border (Figures 2 and 3B) [26]. However, this strain was not included in our study due to the absence of a complete publicly available N gene sequence.

The predominant group of Nepalese viruses of our study belonged to a new Arctic-like sub-clade which we designated as AL-3. This phylogroup shared a common ancestor with the AL-1 sub-clade dating back to 1924 (95% HPD 1871–1968), differing from a previous estimation of as recent as 50 years ago [26]. Within this AL-3 sub-clade, only a single virus V704IRN from the far north-eastern region of Iran, did not originate from Nepal, and was positioned as a stable immediate outlier of the Nepalese group [26], [38]. It may be a hint to its source origin possibly being Central Asian derived Arctic-related RABV. Surprisingly, the N gene phylogeny demonstrated that the group of AL-3 Nepalese isolates, which included samples collected from 1998 to 2011, emerged as recently as the mid-1990's.

The lack of political stability in countries such as Nepal is known to represent a major drawback for the implementation of any programs for disease control. This is particularly true for rabies, which demands a fully integrated approach to achieve its control [41]–[43]. Currently, several critical components to reaching this goal are lacking in Nepal, such as a strong political commitment, the implementation of a coordinated national program for surveillance and control of rabies, dedicated financial resources, and general or widespread public awareness and reporting. Similar factors have been associated with the spread of fox RABV in Europe after the second world war [6], and the high incidence of rabies in the most afflicted regions of the world which encompasses countries presenting with the lowest income, such as sub-Saharan Africa and some countries of south-east Asia [8], [44]. The recent emergence of AL-3 viruses in Nepal further illustrates how the epidemiology of rabies can rapidly change in a country and that a new variant of RABV can rapidly spread in an enzootic country with at least two other genotypic variants already present. The epidemiological fitness of this new lineage and whether it will outcompete the other lineages present in Nepal in the coming years will be of great interest.

A rabies vaccine strain used in Nepal was also phylogenetically characterized and found to be closely related to another vaccine strain from France isolate 93127FRA within the RABV Cosmopolitan clade, thus confirming its historical origin from IPP, France. This vaccine strain had 97% protein sequence similarity to the nucleoprotein of Nepalese viruses in both the Arctic-like and Indian subcontinent clades, whilst the glycoprotein from the vaccine strain had 91–92% similarity to these Nepalese viruses. However cross-protection of isolates belonging to the species RABV in the genus *Lyssavirus* is quite broad and studies have shown vaccine candidates to elicit protective immunity to many varied rabies strains [45], [46].

Physical barriers such as mountains and rivers influence the genetic pool of viruses that circulate in a region as previously highlighted in different settings [5], [6], [47], [48]. Nepal consists of 75 districts which are classified as low, hill and mountain terrains, and is also dominated to the north by the Great Himalayan mountain range. This natural barrier between Nepal and China could be one of the explanations for the absence in Nepal of RABV from the Asian or Cosmopolitan clades [49], although an extensive geographic representation of rabies in Nepal remains difficult to achieve due to the difficulties in transportation and other contributing factors. The Nepalese RABV samples examined in this study originated from seven different districts, all classified within the hill regions of the country. This may explain the uncorrelated distribution of the different RABV lineages in Nepal both within the same, and in multiple districts within the country. In addition, human interference towards facilitating the movement of dogs and other potential rabies hosts to different locations would negate any barriers imposed by moderate terrain. In particular, our study demonstrated the existence of movement of lineage AL-1a related-RABV across the Indian and Nepalese borders.

Evolutionary studies have added to recent knowledge of the Arctic-related clade, especially of the temporal and regional diversification of the Arctic-like sub-clade, and future studies of RABV within the Indian subcontinent could further elucidate the time of introduction of these viruses to Nepal. RABV is a continuing concern in many developing countries such as Nepal, and the increase in knowledge of RABV phylodynamics and epidemiology in these regions would contribute to the development of public awareness and disease control strategies. In particular, our results strongly suggested that the domestic dog is the main reservoir and vector of rabies in Nepal, which is important for the further implementation of surveillance and elimination programs devoted to this disease. Indeed, our observation did not reveal the existence of a sylvatic epidemiological cycle of rabies in Nepal, unlike previously suggested [50], although this observation has to be extended and further confirmed on a larger number of rabies samples from wildlife collected over different parts of the country.

Currently, reported incidence of rabies remains largely underestimated in Nepal. Active surveillance of rabies in domestic and wild animals should be increased and extended to all of the different districts, in an attempt to obtain the most comprehensive picture of the disease epidemiology in the country and to be able to identify spatiotemporal replacement of lineages and the epidemiological causes. Implementation of such a surveillance program represents an achievable goal, through the presence of a large existing network of local veterinary offices (75 district livestock service offices and 999 livestock service centers) throughout the country. However, an effort will be needed to train technical staff in rabies surveillance and on conditions for often challenging sample transportation. In parallel, the incidence of nearly 100 human deaths per year in Nepal has to be more precisely evaluated, as it also appears largely underestimated [51]. Validated laboratory techniques are now available that will contribute to more accurate insights into the incidence and spread of human rabies in developing countries [52]. Such information remains a key step towards the implementation of control measures in endemic countries such as Nepal, contributing to achieving the final goal of elimination of this neglected disease [41], [42].

## Supporting Information

Figure S1 **Maximum likelihood phylogenetic tree based on 92 complete RABV G gene nucleotide sequences.** Branches are labeled with bootstrap values generated with both the neighbor-joining (1,000 bootstrap replications) and maximum likelihood (100 bootstrap replications, number in brackets) algorithms. Bootstrap values are given for selected relevant nodes only. A scale indicating genetic distance is presented by the horizontal bar. Designations of the different RABV phylogroups (clade and sub-clade and lineage) are as indicated, based on previously defined assignments [5], [25]. Isolates for which the complete G gene sequence was obtained in this study are indicated by an asterisk with the viruses from Nepal underscored.
(TIF)

Table S1 **Description of RABV samples and isolates used for phylogenetic analysis.**
(DOCX)
