# Supplementary figures and images for: Recent Emergence and Spread of an Arctic-Related Phylogenetic Lineage of Rabies Virus in Nepal
Source: PLoS Negl Trop Dis. 2013 Nov 21;7(11):e2560. doi: 10.1371/journal.pntd.0002560 (PMC3836727; doi:10.1371/journal.pntd.0002560)

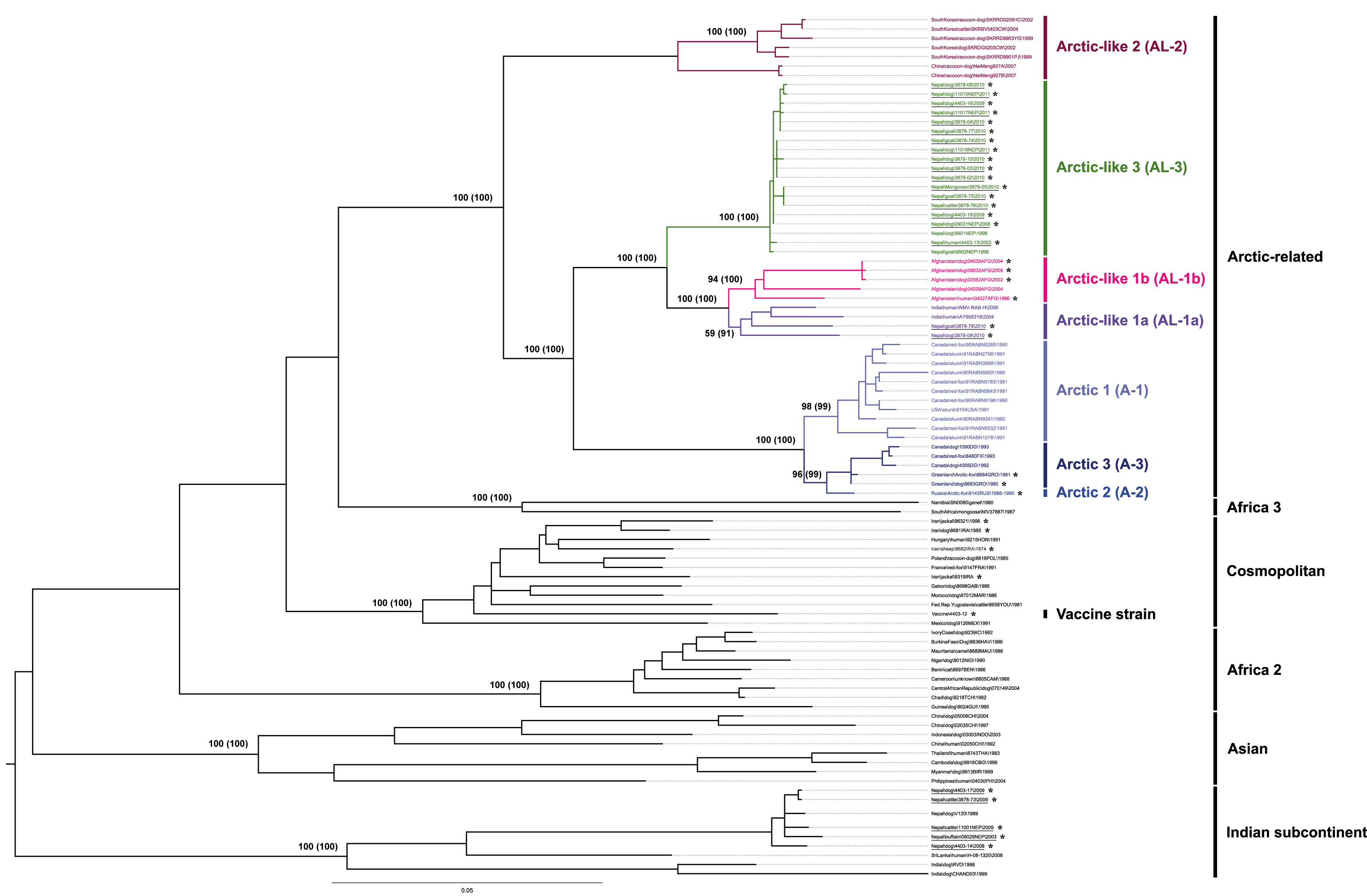

Supplement: Figure S1 — Maximum likelihood phylogenetic tree based on 92 complete RABV G gene nucleotide sequences. Branches are labeled with bootstrap values generated with both the neighbor-joining (1,000 bootstrap replications) and maximum likelihood (100 bootstrap replications, number in brackets) algorithms. Bootstrap values are given for selected relevant nodes only. A scale indicating genetic distance is presented by the horizontal bar. Designations of the different RABV phylogroups (clade and sub-clade and lineage) are as indicated, based on previously defined assignments [5], [25]. Isolates for which the complete G gene sequence was obtained in this study are indicated by an asterisk with the viruses from Nepal underscored. (TIF) [file pntd.0002560.s001.tif]
