# Supplementary material for: Recent Emergence and Spread of an Arctic-Related Phylogenetic Lineage of Rabies Virus in Nepal
Source: PLoS Negl Trop Dis. 2013 Nov 21;7(11):e2560. doi: 10.1371/journal.pntd.0002560 (PMC3836727; doi:10.1371/journal.pntd.0002560)
Supplement: Table S1 — Description of RABV samples and isolates used for phylogenetic analysis. (DOCX) [file pntd.0002560.s002.docx]

Table S1.

Description of RABV samples and isolates used for phylogenetic analysis.

| **Country of origin**  **(region or city)^a^** | **Isolate** | **Year** | **Host species** | **Phylogenetic clade**  **(sub-clade or lineage)** | **GenBank accession no.** | | **References** |
| --- | --- | --- | --- | --- | --- | --- | --- |
|  |  |  |  |  | N gene | G gene |  |
| Afghanistan  (Kabul city) | 04027AFG | 1996 | Human | Arctic-related  (Arctic-like 1b) | EU086162 | JX987719 | This study and [[5](#_ENREF_5)] |
| Afghanistan  (Kabul city) | 02052AFG | 2002 | Dog | Arctic-related  (Arctic-like 1b) | JX987735 | JX987718 | This study and [[5](#_ENREF_5)] |
| Afghanistan  (Kabul city) | 04029AFG | 2004 | Dog | Arctic-related  (Arctic-like 1b) | JX987736 | EU086128 | This study and [[5](#_ENREF_5)] |
| Afghanistan  (Kabul city) | 04035AFG | 2004 | Dog | Arctic-related  (Arctic-like 1b) | GU992304 | JX987720 | This study |
| Afghanistan  (Kabul city) | 09032AFG | 2009 | Dog | Arctic-related  (Arctic-like 1b) | JX987739 | JX987723 | This study |
| Algeria | 9137ALG | 1982 | Dog | Cosmopolitan (Africa 1) | U22643 | - | [[58](#_ENREF_58)] |
| Benin | 8697BEN | 1986 | Cat | Africa 2 | U22485 | FJ545673 | [[16](#_ENREF_16), [58](#_ENREF_58)] |
| Bosnia | 8653YOU | 1986 | Wolf | Cosmopolitan  (Europe) | U42704 | - | [[6](#_ENREF_6)] |
| Burkina Faso | 8636HAV | 1986 | Dog | Africa 2 | U22486 | FJ545672 | [[16](#_ENREF_16), [58](#_ENREF_58)] |
| Cambodia | 9916CBG | 1999 | Dog | Asian | EU086171 | EU086133 | [[5](#_ENREF_5)] |
| Cameroon | 8805CAM | 1988 | Unknown | Africa 2 | U22636 | AF325481 | [[58](#_ENREF_58)] |
| Canada | 90RABN5850 | 1990 | Skunk | Arctic-related  (Arctic-1) | - | U11754 | [[59](#_ENREF_59)] |
| Canada | 90RABN9196 | 1990 | Red fox | Arctic-related  (Arctic-1) | L20676 | U11758 | [[59](#_ENREF_59), [60](#_ENREF_60)] |
| Canada | 90RABN9285 | 1990 | Red fox | Arctic-related  (Arctic-1) | - | U11756 | [[59](#_ENREF_59)] |
| Canada | 90RABN9341 | 1990 | Skunk | Arctic-related  (Arctic-1) | - | U11752 | [[59](#_ENREF_59)] |
| Canada | 6199 | 1991 | Red fox | Arctic-related  (Arctic-1) | U11734 | - | [[59](#_ENREF_59)] |
| Canada | 91RABN0783 | 1991 | Red fox | Arctic-related  (Arctic-1) | L20675 | U11757 | [[59](#_ENREF_59), [60](#_ENREF_60)] |
| Canada | 91RABN1578 | 1991 | Skunk | Arctic-related  (Arctic-1) | L20673 | U11755 | [[59](#_ENREF_59), [60](#_ENREF_60)] |
| Canada | 91RABN2756 | 1991 | Skunk | Arctic-related  (Arctic-1) | L20674 | U11741 | [[59](#_ENREF_59), [60](#_ENREF_60)] |
| Canada | 91RABN3899 | 1991 | Skunk | Arctic-related  (Arctic-1) | - | U11743 | [[59](#_ENREF_59)] |

| Canada | 91RABN5532 | 1991 | Red fox | Arctic-related  (Arctic-1) | - | U11744 | | | [[59](#_ENREF_59)] | | |  |
| --- | --- | --- | --- | --- | --- | --- | --- | --- | --- | --- | --- | --- |
| Canada | 91RABN5643 | 1991 | Red fox | Arctic-related  (Arctic-1) | - | U11746 | | | [[59](#_ENREF_59)] | | |  |
| Canada | 4055DG | 1992 | Dog | Arctic-related  (Arctic-3) | U03770 | U03767 | | | [[61](#_ENREF_61)] | | |  |
| Canada | 1090DG | 1993 | Dog | Arctic-related  (Arctic-3) | U03769 | U03766 | | | [[61](#_ENREF_61)] | | |  |
| Canada | 2244 | 1993 | Red fox | Arctic-related  (Arctic-1) | U11735 | - | | | [[59](#_ENREF_59), [61](#_ENREF_61)] | | |  |
| Canada | 8480FX | 1993 | Red fox | Arctic-related  (Arctic-3) | U03768 | U03765 | | | [[61](#_ENREF_61)] | | |  |
| Central African Rep. | 9228CAF | 1992 | Dog | Cosmopolitan (Africa 1) | U22650 | - | | | [[58](#_ENREF_58)] | | |  |
| Central African Rep. | 070149RCA | 2004 | Dog | Africa 2 | - | FJ545662 | | | [[16](#_ENREF_16)] | | |  |
| Chad | 9218TCH | 1992 | Dog | Africa 2 | U22644 | FJ545683 | | | [[16](#_ENREF_16), [58](#_ENREF_58)] | | |  |
| China | 02050CHI | 1992 | Human | Asian | EU086185 | EU086145 | | | [[5](#_ENREF_5)] | | |  |
| China | 02049CHI | 1993 | Sika deer | Asian | EU086184 | - | | | [[5](#_ENREF_5)] | | |  |
| China | 02035CHI | 1997 | Dog | Asian | EU086174 | EU086136 | | | [[5](#_ENREF_5)] | | |  |
| China | 05008CHI | 2004 | Dog | Asian | EU086189 | EU086149 | | | [[5](#_ENREF_5)] | | |  |
| China  (Inner Mongolia region) | NeiMeng927A | 2007 | Raccoon dog | Arctic-related  (Arctic-like 2) | EU284093 | EU284095 | | | [[57](#_ENREF_57)] | | |  |
| China  (Inner Mongolia region) | NeiMeng927B | 2007 | Raccoon dog | Arctic-related  (Arctic-like 2) | EU652444 | EU284096 | | | [[57](#_ENREF_57)] | | |  |
| Colombia | C01/04  (44148) | 2004 | Dog | Cosmopolitan  (America) | EU086161 | - | | | [[5](#_ENREF_5)] | | |  |
| Czech Republic | 9503TCH | 1995 | Vaccine | Cosmopolitan (vaccine) | GU992319 | - | | | - | | |  |
| Egypt | 8692EGY | 1979 | Human | Cosmopolitan | U22627 | - | | | [[58](#_ENREF_58)] | | |  |
| Estonia | 9342EST | 1991 | Raccoon dog | Cosmopolitan  (Europe) | U43432 | - | | | [[6](#_ENREF_6)] | | |  |
| Ethiopia | 8807ETH | 1987 | Hyena | Cosmopolitan (Africa 1) | U22637 | - | | | [[58](#_ENREF_58)] | | |  |
| Fed. Rep. Yugoslavia  (formerly) | 8658YOU | 1981 | Cattle | Cosmopolitan | U42705 | AF325463 | | | [[6](#_ENREF_6)] | | |  |
| France | 8903FRA | 1989 | Red fox | Cosmopolitan  (Europe) | U42606 | - | | | - | | |  |
| France | 9147FRA | 1991 | Red fox | Cosmopolitan  (Europe) | EU293115 | EU293115 | | | [[15](#_ENREF_15)] | | |  |
| France | 93127FRA | 1993 | Vaccine | Cosmopolitan (vaccine) | GU992320 | - | | | - | | |  |
| Gabon | 8698GAB | 1986 | Dog | Cosmopolitan (Africa 1) | U22630 | AF325470 | | | [[37](#_ENREF_37)] | | |  |
| Germany | 9213ALL | 1991 | Red fox | Cosmopolitan  (Europe) | U42702 | - | | | [[6](#_ENREF_6)] | | |  |
| Germany | 9212ALL | 1991 | Red fox | Cosmopolitan  (Europe) | U22475 | - | | | [[58](#_ENREF_58)] | | |  |
| Guinea | 9024GUI | 1990 | Dog | Africa 2 | U22641 | FJ545680 | | | [[16](#_ENREF_16), [58](#_ENREF_58)] | | |  |
| Greenland | 8683GRO  (8/80) | 1980 | Dog | Arctic-related  (Arctic-3) | JX987745 | JX987730 | | | This study | | |  |
| Greenland | 8684GRO  (31/81) | 1981 | Arctic fox | Arctic-related  (Arctic-3) | U22654 | JX987731 | | | This study | | |  |
| Hungary | 9215HON | 1991 | Human | Cosmopolitan | U43025 | AF325462 | | | [[6](#_ENREF_6)] [[37](#_ENREF_37)] | | |  |
| India | I_107 | - | - | Arctic-related  (Arctic-like 1a) | EF611864 | - | | | [[25](#_ENREF_25)] | | |  |
| India | I_114 | - | - | Arctic-related  (Arctic-like 1a) | EF611863 | - | | | [[25](#_ENREF_25)] | | |  |
| India | I_116 | - | - | Arctic-related  (Arctic-like 1a) | EF611862 | - | | | [[25](#_ENREF_25)] | | |  |
| India | I_123 | - | - | Arctic-related  (Arctic-like 1a) | EF611860 | - | | | [[25](#_ENREF_25)] | | |  |
| India | I_129 | - | - | Arctic-related  (Arctic-like 1a) | EF611859 | - | | | [[25](#_ENREF_25)] | | |  |
| India | I_141 | - | - | Arctic-related  (Arctic-like 1a) | EF611858 | - | | | [[25](#_ENREF_25)] | | |  |
| India | I_145 | - | - | Arctic-related  (Arctic-like 1a) | EF611857 | - | | | [[25](#_ENREF_25)] | | |  |
| India  (Chennai city) | AF374721 | 1999 | Dog | Indian subcontinent | AF374721 | - | | | [[33](#_ENREF_33)] | | |  |
| India | RV61 | 1988 | Human | Arctic-related  (Arctic-like 1a) | AY352493 | - | | | [[55](#_ENREF_55)] | | |  |
| India  (Goa region) | 9702IND | 1997 | Human | Indian subcontinent | EU086191 | - | | | [[5](#_ENREF_5)] | | |  |
| India  (Chennai city) | RVD | 1998 | Dog | Indian subcontinent | - | AY237121 | | | - | | |  |
| India  (Chandigarh city) | CHAND03 | 1999 | Dog | Indian subcontinent | - | AY987478 | | | - | | |  |
| India | AY956319 | 2004 | Human | Arctic-related  (Arctic-like 1a) | AY956319 | AY956319 | | | - | | |  |
| India | NNV-RAB-H | 2006 | Human | Arctic-related  (Arctic-like 1a) | EF437215 | EF437215 | | | [[62](#_ENREF_62)] | | |  |
| Indonesia | 03003INDO | 2003 | Human | Asian | EU086192 | EU086151 | | | [[5](#_ENREF_5)] | | |  |
| Iran  (Shiraz city) | 8682IRA | 1974 | Sheep | Cosmopolitan  (Central Asia) | JX987744 | JX987729 | | | This study | | |  |
| Iran (Kashmar city) | 9319IRA | - | Jackal | Cosmopolitan | JX987746 | JX987732 | | | This study | | |  |
| Iran  (Tehran city) | 8681IRA | 1985 | Dog | Cosmopolitan  (Middle East) | U22482 | JX987728 | | | This study and [[58](#_ENREF_58)] | | |  |
| Iran  (Tehran city) | 8702IRA | 1984 | Wolf | Cosmopolitan  (Middle East) | U22483 | - | | | [[58](#_ENREF_58)] | | |  |
| Iran  (Shahrud city) | 96321IRA | 1996 | Jackal | Cosmopolitan  (Middle East) | JX987748 | JX987734 | | | This study | | |  |
| Iran  (Mashhad city) | V704IRN | 2000 | Sheep | Arctic-related  (Arctic-like 3) | DQ521212 | - | | | [[32](#_ENREF_32)] | | |  |
| Israel | MZ5644 | 1998 | Dog | Cosmopolitan  (Middle East) | DQ837448 | - | | | [[63](#_ENREF_63)] | | |  |
| Ivory Coast | 9026CI | 1990 | Dog | Africa 2 | U22646 |  | | | [[58](#_ENREF_58)] | | |  |
| Ivory Coast | 9239CI | 1992 | Dog | Africa 2 |  | FJ545684 | | | [[16](#_ENREF_16)] | | |  |
| Japan  (Tokyo city) | Komatsugawa | 1940s | Dog | Arctic-related  (Arctic-like 2) | AB178890 | - | | | [[64](#_ENREF_64)] | | |  |
| Japan | Takamen | 1940s | Human | Cosmopolitan | AB178891 | | - | | | | [[64](#_ENREF_64)] |  |
| Kazakhstan | 408s | 1988 | Sheep | Cosmopolitan  (Central Asia) | AY352490 | | - | | | | [[55](#_ENREF_55)] |  |
| Kazakhstan | RV259 | 1988 | Red fox | Cosmopolitan  (Central Asia) | AY352491 | | - | | | | [[55](#_ENREF_55)] |  |
| Mauritania | 8689MAU | 1986 | Camel | Africa 2 | U22489 | | FJ545664 | | | | [[16](#_ENREF_16), [58](#_ENREF_58)] |  |
| Mexico | 9126MEX | 1991 | Dog | Cosmopolitan  (America) | U22477 | | AF325477 | | | | [[58](#_ENREF_58)] |  |
| Mongolia | MGL-36 | 2005 | Dog | Cosmopolitan  (Central Asia) | AB571018 | | - | | | | [[53](#_ENREF_53)] |  |
| Mongolia | MGL-10 | 2006 | Cattle | Cosmopolitan  (Central Asia) | AB570997 | | - | | | | [[53](#_ENREF_53)] |  |
| Mongolia  (Tuv region) | MGL-22 | 2007 | Dog | Arctic-related  (Arctic-like 2) | AB571004 | | - | | | | [[53](#_ENREF_53)] |  |
| Mongolia | MGL-23 | 2008 | Camel | Cosmopolitan  (Central Asia) | AB571005 | | - | | | | [[53](#_ENREF_53)] |  |
| Mongolia | MGL-25 | 2008 | Dog | Cosmopolitan  (Central Asia) | AB571007 | | - | | | | [[53](#_ENREF_53)] |  |
| Morocco | 87012MAR | 1986 | Dog | Cosmopolitan (Africa 1) | U22631 | | AF325467 | | | | [[37](#_ENREF_37), [58](#_ENREF_58)] |  |
| Myanmar | 9913BIR | 1999 | Dog | Asian | EU086165 | | EU086129 | | | | [[5](#_ENREF_5)] |  |
| Namibia | 8708NAM | 1987 | Kudu | Cosmopolitan (Africa 1) | U22632 | | - | | | | [[58](#_ENREF_58)] |  |
| Namibia | SN0080 | 1980 | Genet | Africa 3 | - | | FJ465409 | | | | [[65](#_ENREF_65)] |  |
| Nepal  (supplied by Institut Pasteur, Paris) | 4403-12 | - | Vaccine | Cosmopolitan (vaccine) | JX944594 | | JX944575 | | | | This study |  |
| Nepal  (Kathmandu region) | V120 | 1989 | Dog | Indian subcontinent | - | | AF325489 | | | | [[37](#_ENREF_37)] |  |
| Nepal  (Kathmandu region) | 9901NEP | 1998 | Dog | Arctic-related  (Arctic-like 3) | EU086196 | | EU086153 | | | | [[5](#_ENREF_5)] |  |
| Nepal  (Kathmandu region) | 9902NEP | 1998 | Goat | Arctic-related  (Arctic-like 3) | EU086197 | | EU086154 | | | | [[5](#_ENREF_5)] |  |
| Nepal  (Kathmandu region) | 9903NEP | 1998 | Mongoose | Arctic-related  (Arctic-like 3) | EU086198 | | - | | | | [[5](#_ENREF_5)] |  |
| Nepal (Pokhara region) | 09029NEP^b^ | 2003 | Buffalo | Indian subcontinent | JX987737 | | JX987721 | | | | This study |  |
| Nepal (Lalitpur region) | 4403-13  (09030NEP)^b^ | 2003 | Human | Arctic-related  (Arctic-like 3) | JX944565 | | JX944584 | | | | This study |  |
| Nepal (Kathmandu region) | 09031NEP^b^ | 2008 | Dog | Arctic-related  (Arctic-like 3) | JX987738 | | JX987722 | | | | This study |  |
| Nepal (Kathmandu region) | 4403-14^b^ | 2008 | Dog | Indian subcontinent | JX944595 | | JX944576 | | | | This study |  |
| Nepal (Lalitpur region) | 11001NEP^b^ | 2009 | Cattle | Indian subcontinent | JX987740 | | JX987724 | | | | This study |  |
| Nepal (Pokhara region) | 3878-73^b^  (11003NEP) | 2009 | Cattle | Indian subcontinent | JX944569 | | JX944588 | | | | This study |  |
| Nepal (Dhading region) | 4403-16^b^ | 2009 | Dog | Arctic-related  (Arctic-like 3) | JX944596 | | JX944577 | | | | This study |  |
| Nepal (Pokhara region) | 4403-17^b^ | 2009 | Dog | Indian subcontinent | JX944597 | | JX944578 | | | | This study |  |
| Nepal (Lalitpur region) | 4403-19^b^ | 2009 | Dog | Arctic-related  (Arctic-like 3) | JX944598 | | | JX944579 | | This study | | |
| Nepal (Pokhara region) | 3878-2^b^  (11002NEP) | 2010 | Dog | Arctic-related  (Arctic-like 3) | JX944566 | | | JX944585 | | This study | | |
| Nepal (Kathmandu region) | 3878-03^b^ | 2010 | Dog | Arctic-related  (Arctic-like 3) | JX944599 | | | JX944580 | | This study | | |
| Nepal (Bhaktapur region) | 3878-04^b^ | 2010 | Dog | Arctic-related  (Arctic-like 3) | JX944600 | | | JX944581 | | This study | | |
| Nepal (Pokhara region) | 3878-05^b^ | 2010 | Mongoose | Arctic-related  (Arctic-like 3) | JX944601 | | | JX944582 | | This study | | |
| Nepal (Kathmandu region) | 3878-08^b^ | 2010 | Dog | Arctic-related  (Arctic-like 3) | JX944602 | | | JX944583 | | This study | | |
| Nepal (Kathmandu region) | 3878-09^b^  (11009NEP) | 2010 | Dog | Arctic-related  (Arctic-like 1a) | JX944567 | | | JX944586 | | This study | | |
| Nepal (Kathmandu region) | 3878-10^b^  (11013NEP) | 2010 | Dog | Arctic-related  (Arctic-like 3) | JX944568 | | | JX944587 | | This study | | |
| Nepal (Tanahu region) | 3878-74^b^  (11004NEP) | 2010 | Goat | Arctic-related  (Arctic-like 3) | JX944570 | | | JX944589 | | This study | | |
| Nepal (Pokhara region) | 3878-75^b^  (11005NEP) | 2010 | Goat | Arctic-related  (Arctic-like 3) | JX944571 | | | JX944590 | | This study | | |
| Nepal (Pokhara region) | 3878-76^b^  (11006NEP) | 2010 | Cattle | Arctic-related  (Arctic-like 3) | JX944572 | | | JX944591 | | This study | | |
| Nepal (Bhaktapur region) | 3878-77^b^ (11007NEP) | 2010 | Goat | Arctic-related  (Arctic-like 3) | JX944573 | | | JX944592 | | This study | | |
| Nepal (Makwanpur region) | 3878-78^b^ (11008NEP) | 2010 | Goat | Arctic-related  (Arctic-like 1a) | JX944574 | | | JX944593 | | This study | | |
| Nepal (Kathmandu region) | 11015NEP^b^ | 2011 | Dog | Arctic-related  (Arctic-like 3) | JX987741 | | | JX987725 | | This study | | |
| Nepal (Kathmandu region) | 11016NEP^b^ | 2011 | Dog | Arctic-related  (Arctic-like 3) | JX987742 | | | JX987726 | | This study | | |
| Nepal (Kathmandu region) | 11017NEP^b^ | 2011 | Dog | Arctic-related  (Arctic-like 3) | JX987743 | | | JX987727 | | This study | | |
| Niger | 9012NIG | 1990 | Dog | Africa 2 | U22640 | | | FJ545678 | | [[16](#_ENREF_16), [58](#_ENREF_58)] | | |
| Pakistan | 196p | 1990 | Cow | Arctic-related  (Arctic-like 1b) | AY352495 | | | - | | [[55](#_ENREF_55)] | | |
| Pakistan | 277p | 1990 | Goat | Arctic-related  (Arctic-like 1b) | AY352496 | | | - | | [[55](#_ENREF_55)] | | |
| Philippines | 04030PHI | 2004 | Human | Asian | EU086205 | | | EU086155 | | [[5](#_ENREF_5)] | | |
| Poland | 8618POL | 1985 | Raccoon dog | Cosmopolitan  (Europe) | U22840 | | | AF325464 | | [[37](#_ENREF_37), [58](#_ENREF_58)] | | |
| Russia  (Tuva region) | RV250 | - | Rodent | Arctic-related  (Arctic-2) | AY352480 | | | - | | [[55](#_ENREF_55)] | | |
| Russia | RV1590 | - | Human | Cosmopolitan  (Central Asia) | AY352472 | | | - | | [[55](#_ENREF_55)] | | |
| Russia  (Yakutia region) | SG10 | 1950-1960 | Arctic fox | Arctic-related  (Arctic-3) | EF611838 | | | - | | [[25](#_ENREF_25)] | | |
| Russia  (Yakutia region) | SG15 | 1950-1960 | Arctic fox | Arctic-related  (Arctic-2) | EF611840 | | | - | | [[25](#_ENREF_25)] | | |
| Russia  (Yakutia region) | SG92 | 1950-1960 | Arctic fox | Arctic-related  (Arctic-2) | EF611835 | | | - | | [[25](#_ENREF_25)] | | |
| Russia  (Yakutia region) | SG12 | 1950-1960 | Arctic fox | Arctic-related  (Arctic-2) | EF611837 | | | - | | [[25](#_ENREF_25)] | | |
| Russia  (Yakutia region) | SG91 | 1950-1960 | Arctic fox | Arctic-related  (Arctic-2) | EF611834 | - | | | [[25](#_ENREF_25)] | | |  |
| Russia  (Chita region) | 304c | 1977 | Steppe fox | Arctic-related  (Arctic-like 2) | AY352459 | - | | | [[55](#_ENREF_55)] | | |  |
| Russia  (Chita region) | 248c | 1977 | Steppe fox | Arctic-related  (Arctic-like 2) | AY352460 | - | | | [[55](#_ENREF_55)] | | |  |
| Russia  (Chabarovsk region) | 857r | 1980 | Raccoon dog | Arctic-related  (Arctic-like 2) | AY352458 | - | | | [[55](#_ENREF_55)] | | |  |
| Russia  (Southern Siberia) | 994_dog | 1980 | Dog | Arctic-related  (Arctic-like 2) | EF611868 | - | | | [[25](#_ENREF_25)] | | |  |
| Russia  (Yakutia region) | 483a | 1986 | Arctic fox | Arctic-related  (Arctic-2) | AY352487 | - | | | [[55](#_ENREF_55)] | | |  |
| Russia  (Yakutia region) | SG21 | 1987 | Arctic fox | Arctic-related  (Arctic-2) | EF611828 | - | | | [[25](#_ENREF_25)] | | |  |
| Russia  (Yakutia region) | SG22 | 1987 | Arctic fox | Arctic-related  (Arctic-2) | EF611831 | - | | | [[25](#_ENREF_25)] | | |  |
| Russia | 686cow | 1987 | Cow | Cosmopolitan  (Central Asia) | AY352482 | - | | | [[55](#_ENREF_55)] | | |  |
| Russia  (Yakutia region) | 9141RUS (RV251.748) | 1988 | Arctic fox | Arctic-related  (Arctic-2) | U22656 | - | | | This study and [[58](#_ENREF_58)] | | |  |
| Russia  (Yakutia region) | 9143RUS (RV294.994) | 1988-90 | Arctic fox | Arctic-related  (Arctic-2) | JX987747 | JX987733 | | | This study | | |  |
| Russia | 765w | 1989 | Wolf | Cosmopolitan  (Central Asia) | AY352483 | - | | | [[55](#_ENREF_55)] | | |  |
| Russia  (Yakutia region) | 3510w | 1995 | Wolf | Arctic-related  (Arctic-2) | AY352486 | - | | | [[55](#_ENREF_55)] | | |  |
| Russia | 3561d | 1996 | Dog | Cosmopolitan  (Central Asia) | AY352481 | - | | | [[55](#_ENREF_55)] | | |  |
| Russia  (Yakutia region) | 743a | 1988 | Arctic fox | Arctic-related  (Arctic-2) | AY352488 | - | | | [[55](#_ENREF_55)] | | |  |
| Russia  (Krasnoyarsk region) | RVHK | 1998 | Human | Arctic-related  (Arctic-3) | AY352462 | - | | | [[55](#_ENREF_55)] | | |  |
| Saudi Arabia | 8706ARS | 1987 | Fox | Cosmopolitan  (Middle East) | EU086163 | - | | | [[5](#_ENREF_5)] | | |  |
| South Africa | 8721AFS | 1981 | Human | Cosmopolitan (Africa 1) | U22633 | - | | | [[58](#_ENREF_58)] | | |  |
| South Africa | 1500AFS | 1987 | Mongoose | Africa 3 | U22628 | - | | | [[58](#_ENREF_58)] | | |  |
| South Africa | NIV378/87 | 1987 | Mongoose | Africa 3 | - | AF325485 | | | [[37](#_ENREF_37)] | | |  |
| South Korea  (Paju-si region) | SKRRD9901PJ | 1999 | Raccoon dog | Arctic-related  (Arctic-like 2) | DQ076123 | DQ076103 | | | [[54](#_ENREF_54)] | | |  |
| South Korea  (Yanggu-gun region) | SKRRD9903YG | 1999 | Raccoon dog | Arctic-related  (Arctic-like 2) | DQ076131 | DQ076099 | | | [[54](#_ENREF_54)] | | |  |
| South Korea  (Cheorwon-gun region) | SKRDG0203CW | 2002 | Dog | Arctic-related  (Arctic-like 2) | DQ076124 | DQ076104 | | | [[54](#_ENREF_54)] | | |  |
| South Korea  (Hwacheon-gun region) | SKRRD0205HC | 2002 | Raccoon dog | Arctic-related  (Arctic-like 2) | DQ076127 | DQ076096 | | | [[54](#_ENREF_54)] | | |  |
| South Korea  (Cheorwon-gun region) | SKRBV0403CW | 2004 | Cattle | Arctic-related  (Arctic-like 2) | DQ076129 | DQ076095 | | | [[54](#_ENREF_54)] | | |  |
| South Korea  (Chuncheon city) | KRC5_04 | 2004 | Dog | Arctic-related  (Arctic-like 2) | AY730597 | - | | | [[56](#_ENREF_56)] | | |  |
| South Korea  (Hongcheon region) | KRH2-04 | 2004 | Raccoon dog | Arctic-related  (Arctic-like 2) | AY730595 | - | | | [[56](#_ENREF_56)] | | |  |
| Sri Lanka  (Colombo city) | 1294 | 1986 | Dog | Indian subcontinent | AY138549 | - | | | [[36](#_ENREF_36)] | | |  |
| Sri Lanka  (Pannipitiya region) | SRL1032 | 1996 | Jackal | Indian subcontinent | AB041964 | - | | | [[29](#_ENREF_29)] | | |  |
| Sri Lanka  (Ragama region) | SRL1036 | 1996 | Human | Indian subcontinent | AB041965 | - | | | [[29](#_ENREF_29)] | | |  |
| Sri Lanka  (Seeduwa region) | SRL1060 | 1996 | Dog | Indian subcontinent | AB041966 | - | | | [[29](#_ENREF_29)] | | |  |
| Sri Lanka  (Naranhenpita region) | SRL1077 | 1996 | Mongoose | Indian subcontinent | AB041967 | - | | | [[29](#_ENREF_29)] | | |  |
| Sri Lanka  (Waskaduwa region) | SRL1143 | 1996 | Cat | Indian subcontinent | AB041968 | - | | | [[29](#_ENREF_29)] | | |  |
| Sri Lanka  (Maharagama region) | SRL1145 | 1996 | Water buffalo | Indian subcontinent | AB041969 | - | | | [[29](#_ENREF_29)] | | |  |
| Sri Lanka | 5657 | 2001 | Bovine | Indian subcontinent | AY138550 | - | | | [[36](#_ENREF_36)] | | |  |
| Sri Lanka  (Gampaha city) | H-08-1320 | 2008 | Human | Indian subcontinent | AB569299 | AB569299 | | | [[34](#_ENREF_34)] | | |  |
| Sri Lanka  (Galle city) | H-219-08 | 2008 | Human | Indian subcontinent | AB638767 | - | | | [[34](#_ENREF_34)] | | |  |
| Sri Lanka  (Matara city) | H-1218-08 | 2008 | Human | Indian subcontinent | AB638768 | - | | | [[34](#_ENREF_34)] | | |  |
| Sri Lanka  (Moneragala city) | H-1281-08 | 2008 | Human | Indian subcontinent | AB638769 | - | | | [[34](#_ENREF_34)] | | |  |
| Sri Lanka (Matale city) | H-15-09 | 2009 | Human | Indian subcontinent | AB638770 | - | | | [[34](#_ENREF_34)] | | |  |
| Sri Lanka  (Kegalle city) | H-156-09 | 2009 | Human | Indian subcontinent | AB638771 | - | | | [[34](#_ENREF_34)] | | |  |
| Sri Lanka (Kalutara city) | H-1366-09 | 2009 | Cat | Indian subcontinent | AB638772 | - | | | [[34](#_ENREF_34)] | | |  |
| Tanzania | 9224TAN | 1992 | Wild dog | Cosmopolitan (Africa 1) | U22648 | - | | | [[58](#_ENREF_58)] | | |  |
| Thailand | 8743THA | 1983 | Human | Asian | EU293121 | EU293121 | | | [[15](#_ENREF_15)] | | |  |
| USA (Alaska) | 1420 | - | Red fox | Arctic-related  (Arctic-2) | AY352499 | - | | | [[55](#_ENREF_55)] | | |  |
| USA (Alaska) | 1421 | 1988 | Red fox | Arctic-related  (Arctic-3) | AY352500 | - | | | [[55](#_ENREF_55)] | | |  |
| USA (Alaska) | 4795 | 1988 | Dog | Arctic-related  (Arctic-2) | AY352498 | - | | | [[55](#_ENREF_55)] | | |  |
| USA  (Montana) | 9104USA (CRBIP8.32) | 1991 | Skunk | Arctic-related  (Arctic-1) | GU992317 | JX987749 | | | - | | |  |
| USA  (Montana) | 9105USA | 1990 | Red fox | Arctic-related  (Arctic-1) | U22655 | - | | | [[58](#_ENREF_58)] | | |  |
| USA (Alaska) | A0903 | 2006 | Dog | Arctic-related  (Arctic-4) | EF611855 | - | | | [[25](#_ENREF_25)] | | |  |
| USA (Alaska) | A0904 | 2006 | Red fox | Arctic-related  (Arctic-2) | EF611854 | - | | | [[25](#_ENREF_25)] | | |  |
| USA (Alaska) | A0906 | 2006 | Arctic fox | Arctic-related  (Arctic-2) | EF611856 | - | | | [[25](#_ENREF_25)] | | |  |
| USA (Alaska) | A6091 | 2006 | Red fox | Arctic-related  (Arctic-3) | EF611849 | - | | | [[25](#_ENREF_25)] | | |  |
| USA (Alaska) | A7027 | 2007 | Red fox | Arctic-related  (Arctic-4) | EF611843 | - | | | [[25](#_ENREF_25)] | | |  |

| USA (Alaska) | A7032 | 2007 | Red fox | Arctic-related  (Arctic-3) | EF611850 | - | [[25](#_ENREF_25)] |
| --- | --- | --- | --- | --- | --- | --- | --- |
| USA (Alaska) | A7033 | 2007 | Red fox | Arctic-related  (Arctic-3) | EF611845 | - | [[25](#_ENREF_25)] |

^a^Locality or city is indicated when available and only for selected isolates (including strains previously unpublished or isolates belonging to the Arctic-related or Indian subcontinent clades, and used in part for the achievement of Figure 1).

^b^Nepalese strains collected in this study and presented in the Figure 1B.
